# Management of epithelial ovarian cancer in sub-Saharan Africa: a survey of practicing physicians

**DOI:** 10.3332/ecancer.2026.2125

**Published:** 2026-05-20

**Authors:** Ya Haddy Sallah, Verna Vanderpuye, Rahel Ghebre, Khadija Warfa, Alex Mutombo Baleka, Hannah Naa Gogwe Ayettey Anie, Pa Omadou Sallah, Esayas Berhanu Enoro, Namugga Jane, Rose Anorlu

**Affiliations:** 1 Department of Medicine, Memorial Sloan Kettering Cancer Center, New York, NY 10065, USA; 2 National Radiotherapy Oncology and Nuclear Medicine Centre, Korle Bu Teaching Hospital, Accra, Ghana; 3 University of Minnesota Medical School and Masonic Cancer Center, Minneapolis, MN 55455, USA; 4 Aga Khan University Hospital, Nairobi, Kenya; 5 Kinshasa University﻿﻿ Hospital, Democratic Republic of Congo; 6 Johns Hopkins University School of Advanced International Studies, Washington, DC 20001, USA; 7 College of Health Science, Addis Ababa University, Addis Ababa, Ethiopia; 8 Mulago Specialized Women and Neonatal Hospital, Kampala, Uganda; 9 College of Medicine, University of Lagos, Lagos University Teaching Hospital, Lagos, Nigeria

**Keywords:** ovarian cancer, sub-Saharan Africa, diagnosis, management, disparities

## Abstract

The purpose of this study was to evaluate current practices in the management of epithelial ovarian cancer (OC) in sub-Saharan Africa (SSA). A 27-question survey was distributed to SSA-based physicians who managed OC and were members of the African Organisation for Research and Training in Cancer (AORTIC) via Research Electronic Data Capture. Questions evaluated diagnostics, imaging, surgical capacity, guideline adherence, access and barriers to systemic therapies, genetic testing and palliative care. Survey responses from 58 providers in 25 SSA countries were analysed. Clinical/medical oncologists and gynaecologic oncologists made up 52% and 36% of the respondents, respectively. Harmonised guidelines for SSA and the European Society of Medical Oncology guidelines were the most commonly used guidelines among respondents. Surgeries were performed by gynaecologic oncologists (53%) and general gynaecologists (38%). Complete cytoreductive surgery was the primary surgical goal for 38% of the respondents. Lymphadenectomy was routinely performed in 34% of patients with early-stage disease. The majority of systemic therapies were prescribed by clinical/medical oncologists (76%). Platinum/taxane doublet chemotherapy was the prevalent choice for adjuvant (90%) and neoadjuvant indications (83%). Single-agent gemcitabine was preferred for treating platinum-resistant disease (55%). Bevacizumab was prescribed by 33% of the providers. BRCA testing was limited. Drugs that target BRCA-positive tumours were prescribed by 12% of the respondents. Referral to routine palliative care services for advanced disease was performed by 41% of the respondents. The survey results demonstrate areas of guideline-concordant care for OC in SSA despite several challenges, including limited subspecialty surgical capacity and highlight important remaining gaps. To improve OC outcomes in SSA, increasing gynaecologic oncology capacity, promoting and adopting context-specific guidelines and supporting implementation-focused research require prioritisation.

## Background

Ovarian cancer (OC) is the eighth leading cause of cancer-related mortality among women worldwide [1]. Patients often present with advanced-stage disease due to prolonged non-specific symptoms [2]. In sub-Saharan Africa (SSA), the reported age-standardised mortality rates of OC range from 3.3 to 4.4 per 100,000 person-years, which is comparable to the global average of 4.2 per 100,000 person-years [1]. However, institution-level estimates of 5-year OC survival in Africa have been as low as 38%, whereas the estimated global 5-year survival rate is 46% (3). The incidence of OC in Africa has steadily increased over the past 20 years, which represents an urgent call for action to improve clinical outcomes [3–5].

The National Cancer Control (NCCN) harmonised guidelines for SSA and the American Society of Clinical Oncology (ASCO) resource-stratified guidelines recommend routine imaging with ultrasound (US), computed tomography (CT) and/or magnetic resonance imaging (MRI) if available. Initial measurements of the CA-125 tumour marker are recommended by both guidelines [6, 7]. ASCO resource-stratified guidelines prefer CT-guided biopsy or laparoscopy over laparotomy and open biopsy [7].

Neoadjuvant chemotherapy (NACT) followed by interval debulking surgery (IDS) for advanced-stage disease has noninferior disease-free and overall survival outcomes, with lower morbidity rates than primary debulking surgery (PDS); however, the optimal treatment sequence remains controversial and is the subject of ongoing clinical trials [8]. NACT followed by surgical debulking is recommended for patients with a low likelihood of optimal cytoreduction with PDS [8]. OC surgery with optimal debulking performed by gynaecologic oncologists is recommended even in low-resource regions [7]. However, studies have shown limitations in the number of skilled gynaecology oncology surgeons in SSA with high volume experience in performing radical surgeries of involved organs, lymphadenectomies and obtaining negative margins in patients with cervical cancer, a related gynaecologic malignancy [9].

In line with NCCN harmonised and ASCO resource-stratified guideline recommendations, combination platinum-based chemotherapy remains the standard care for first-line therapy except in those with poor performance status or elderly patients, where single-agent platinum is recommended [6, 7]. In platinum-resistant recurrent disease, there are multiple single-agent chemotherapy options in SSA-specific resource-stratified guidelines [6]. Over the past decade, several advances have been made in the management of OC, including the use of poly (ADP‒ribose) polymerase inhibitors (PARPis) for *BRCA*-mutated or homologous recombination-deficient tumours and antiangiogenic agents such as bevacizumab [10–12]. Some of these targeted therapies require molecular testing techniques such as next-generation sequencing, which are not readily available in low- or lower-middle-income countries (LMICs) [13]. Historically, the prohibitive cost of drugs, out-of-pocket payments, insufficient surgical capacity, poor access to advanced diagnostics and presumed challenges in managing toxicities from systemic therapies have limited the implementation of cancer care guidelines in many LMICs [14].

Survey-based studies of professional oncology societies in high-income countries (HICs) have shown significant variations in practice patterns of OC management [15–18]. To the best of our knowledge, the clinical management practices of providers treating OC in SSA have not been systematically examined at the time of conducting this research, highlighting a critical gap in the literature. This study aimed to determine the prevailing practice patterns in the management of OC in SSA, including initial diagnosis and staging, surgical appropriateness, systemic therapies, genetic testing and palliative care, by leveraging the robust professional network of African Organisation for Research and Training in Cancer (AORTIC), given the lack of a centralised database of treating providers in SSA.

## Methods

Institutional review board approval was obtained from the Korle-Bu Teaching Hospital in Accra, Ghana (KBTH-IRB approval number: 0001652/2023). The survey was generated via Research Electronic Data Capture software. Survey questions were developed through a literature review in tandem with consultation of practitioners affiliated with the AORTIC and study co-authors, several of whom have contributed to the NCCN and ASCO resource-stratified OC management guidelines for SSA and resource-constrained settings (VV, RG, EBO, JN). The survey was available in English and French, two widely spoken languages in SSA. This study followed the principles of the Declaration of Helsinki. Participants received an informed consent document that stated that by completing the survey, they were consenting to participation. The survey responses were anonymous, with no identifying information collected that could be used to identify participants during or after the data collection. The survey was tested and internally validated by study authors (YHS VV, AB, RG), who are oncology providers with experience in the management of OC. No incentives were provided to participate in this survey.

The structured online survey consisted of a total of 27 questions (Appendix I). Twenty-five of these questions were multiple-choice and two questions required free-text responses. Broadly, these questions were divided into five categories: demographics, diagnosis and staging, surgery, systemic therapy choices and palliative care. The final survey was distributed via the AORTIC mailing list between December 2023 and February 2024, for a total of 80 days. This distribution strategy, which has been used in surveys conducted in other regions, precluded the determination of the total number of providers who received the survey; therefore, a response rate could not be calculated. The authors also shared the survey link with relevant professional contacts via a snowball sampling approach. Two follow-up reminders were sent to encourage participation. Responses from participants who reported that they were not directly involved in OC management were automatically terminated. The survey was queried at 1-week intervals. º

Descriptive statistics, including counts and percentages, were used to summarise the data. For select questions, participants were allowed to select more than response and total percentages for these questions did not equate to 100. Countries were categorised into income groups on the basis of the 2021 World Bank classification. Survey respondents were asked to identify the main challenges faced in managing OC with a free-text response. The responses were summarised into major themes and subthemes. Graphs and visualisations were developed via the R statistical environment version (R version 4.3.4, R Foundation for Statistical Computing, Vienna, Austria) and Tableau (Tableau Public 2024.1, Tableau Software, Mountain View, California).

## Results

### Respondent characteristics

**Figure 1.**
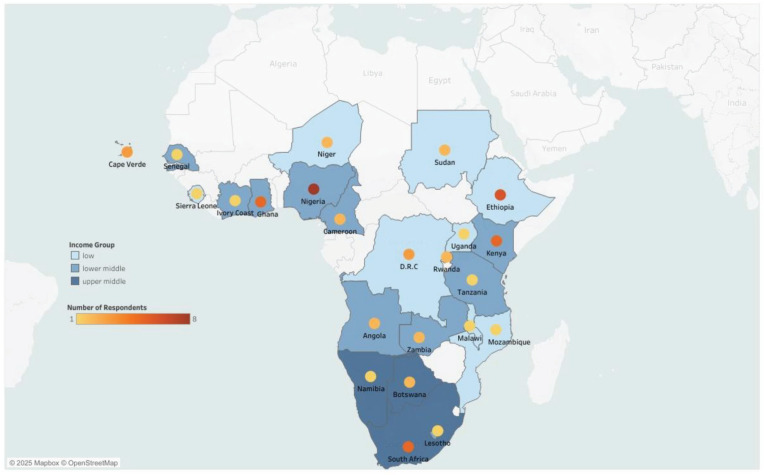
Geographic distribution of survey respondents by WHO income group and number of respondents.

A total of 58 respondents participated, representing 25 out of the 46 countries in SSA (Figure 1). The largest number of respondents came from Nigeria (*n* = 8), followed by Ethiopia (*n* = 6), South Africa (*n* = 5), Kenya (*n* = 5) and Ghana (*n* = 4). Most participants were based in West Africa (33%) and worked in low-income countries (50%). Approximately 14% and 34% of the participants practiced in upper-middle- and LMICs, respectively. Among the 50 providers who reported their institution, 78% practiced in public hospitals, 16% in private hospitals and 6% in hospitals with private–public partnerships.

The respondents were medical/clinical oncologists (52%), gynaecologic oncologists (36%), gynaecologists (9%) and surgical oncologists (3%).

Government insurance was the primary method of financing OC care (59%).

Most respondents used published guidelines for OC management (92%). The NCCN Harmonised Guidelines for SSA and the European Society of Medical Oncology (ESMO) Guidelines were the most popular. Only 22% of the respondents used regional and national cancer guidelines.

### Diagnosis and staging

CT was the most commonly used imaging modality (62%) as recommended by the ASCO resource-stratified guidelines if available in enhanced-resource settings [6], followed by US (60%) and MRI (36%). Approximately 67% of providers reported that biopsies were routinely performed at their institutions to confirm OC diagnosis before any intervention. Resource stratified guidelines recommend minimally invasive approaches for diagnosis if undertaken [6]. However, most biopsies were open (44%). Imaging-guided biopsies were the second most common method (26%), followed by cytology of ascitic fluid (23%) and laparoscopy (15%). ASCO resource-stratified guidelines recommend that a CA-125 or carcinoembryonic antigen level should be checked if a histologic-based diagnosis cannot be made to support the diagnosis of OC [6]. The tumour marker CA-125 level was always checked at diagnosis by 78% of the respondents, whereas 19% reported that CA-125 levels were sometimes checked at diagnosis, and 3% of the respondents reported that CA-125 levels were rarely or never obtained before interventions. Twenty-two percent of participants reported the use of a scoring system such as the Fagotti score to predict the likelihood of optimal cytoreduction during surgery.

The ASCO resource-stratified guidelines for OC recommend offering genetic testing only if actionable next steps, such as genetic counselling and mutation-directed targeted therapy, are available [6]. In our cohort, genetic testing and counseling were sometimes performed by 21% of respondents, rarely performed by 24% and never performed by 17% of respondents. Additionally, 34% of the respondents reported that genetic testing for OC was not available in their country.

### Surgery

**Figure 2.**
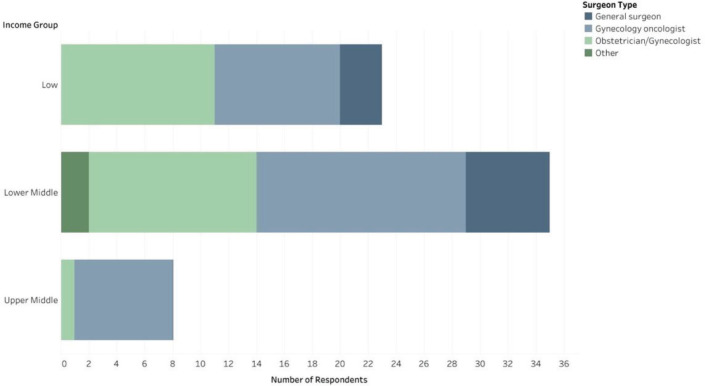
Providers who perform cytoreductive surgery by country income level.

The NCCN harmonised guidelines for SSA and ASCO resource-stratified guidelines recommend that cytoreductive surgery should be performed by gynaecologic oncologists (preferred) if available or general surgeons with high-volume experience in cancer surgery [6, 7]. Cytoreductive surgery was most commonly performed by gynaecologic oncologists (53%) and general obstetricians/gynaecologists (38%) (Figure 2). Complete cytoreduction with no residual disease was reported as the surgical goal at their institution by 38% of the respondents. Both guidelines recommend resection of all visible tumour with <1 cm and, ideally, no gross residual disease [6, 7]. Optimal cytoreductive surgery with <1 cm of residual disease was reported as the institutional goal by 47% of the respondents; 16% of the respondents reported that >1 cm of residual disease was the goal of surgery at their institution.

According to 34% of the respondents, routine systematic lymphadenectomy was performed for early-stage disease, whereas 22% reported that it was performed only for advanced-stage disease. Approximately 12% of providers reported that all patients undergo lymphadenectomy, and 29% reported that it was not available at their institution. Sixty percent of the respondents reported that general surgeons assisted in performing liver, splenic or bowel resections when indicated. NCCN harmonised guidelines for SSA recommend consideration of secondary cytoreductive surgery in patients with recurrent OC more than 6 to 12 months after completion of initial chemotherapy, particularly in those with limited disease amenable to complete resection with no ascites [6]. Secondary cytoreductive surgery for low-volume recurrent disease was reported as a routine practice in their institution by 22% of the respondents.

### Systemic therapy

**Figure 3.**
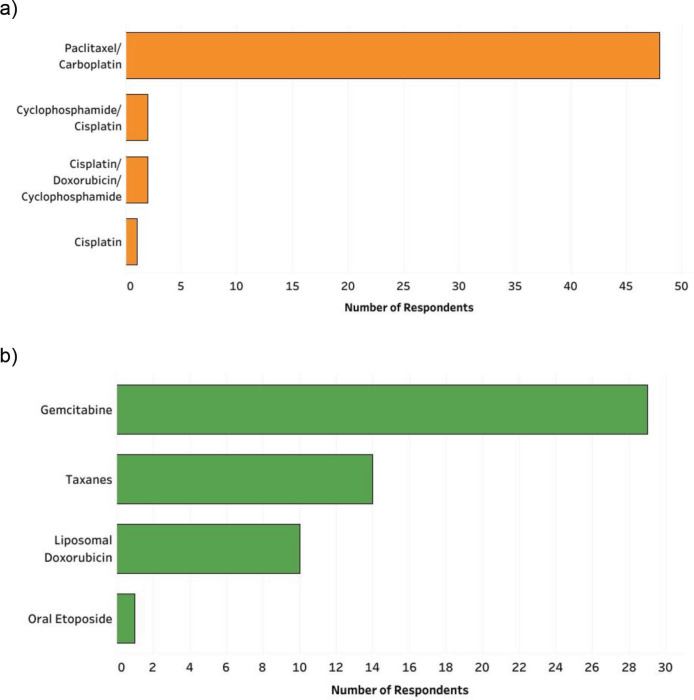
Preferred chemotherapy regimens for (a): NACT and (b): Platinum-resistant recurrent OC.

Chemotherapy was predominantly prescribed by clinical/medical oncologists (76%). The majority of respondents (83%) reported platinum/paclitaxel doublet chemotherapy as the preferred first-line NACT in accordance with NCCN harmonised and ASCO resource-stratified guidelines [6, 7] (Figure 3a) and adjuvant therapy (90%). Few respondents reported using alternative platinum doublets or single-agent platinum as a first-line regimen, which is recommended by NCCN guidelines for patients older than 70 or with significant medical comorbidities [6]. NACT followed by IDS for advanced disease was the preferred approach for 64% of the respondents. NCCN harmonised guidelines recommend 3–4 cycles of NACT before IDS [6]. In our study, the most commonly reported number of cycles of neoadjuvant therapy was three (39%), with responses ranging from three to six. Among respondents who reported an absolute number of cycles, the mean number of cycles of NACT was 4.07 (SD = 1.25).

In patients with recurrent platinum-resistant disease, single-agent gemcitabine (55%) was the preferred regimen, followed by taxanes (24%), liposomal doxorubicin (17%) and oral etoposide (2%) (Figure 3b).

ASCO resource-stratified guidelines presume that targeted therapies such as PARP inhibitors and bevacizumab are not available in limited resource settings. However, in resource-enhanced settings, maintenance bevacizumab is recommended if available [7]. Approximately 65% of the respondents reported that bevacizumab was not available in their institution. Fewer than 10% of the respondents prescribed bevacizumab for maintenance therapy. PARP inhibitors were generally unavailable (60%). Only 12% of the respondents reported routinely prescribing them to patients harbouring *BRCA* mutations.

### Palliative and supportive care

Early palliative care involvement in advanced OC is recommended by ASCO resource-stratified guidelines [7]. When asked how often they referred patients with advanced OC to palliative care, 41% reported that they usually did, 43% sometimes referred patients and 5% reported the unavailability of palliative care services.

### Challenges in OC management

The major themes, subthemes and challenges faced in the management of OC are summarised in Table 1.

## Discussion

This survey-based study of oncology providers, including medical/clinical oncologists, gynaecologic oncologists and general gynaecologists from 25 of the 46 countries in SSA, highlights current practices of OC and major challenges, including surgical capacity, late presentation at diagnosis, high cost of care and lack of access to novel therapies for OC.

Our study found low use of national or regionally developed guidelines among SSA oncology practitioners for OC management compared with resource-stratified guidelines such as the NCCN and ASCO guidelines. Resource-stratified guidelines are intended to provide a flexible approach that considers resource constraints; however, they often rely on evidence adapted from HICs [19, 20]. National cancer care guidelines may be more tailored to a country’s specific local context, healthcare infrastructure and cultural factors that influence care delivery [21]. There is a growing call to incorporate context-specific research derived from LMICs rather than adapting resource-stratified guidelines from HICs [19]. Establishing context-specific guidelines for the management of OC is particularly important to reduce treatment abandonment, relieve financial strain, minimise provider burnout and avoid placing unnecessary pressure on fragile health systems.

The availability of CT scans, as opposed to MRI, has significantly increased in many SSA countries, especially in urban centers and academic hospitals where oncologists, radiologist and surgeons are more likely to be based and where infrastructure investment is relatively higher [22]. In our study, CT scans emerged as the imaging modality of choice for initial diagnostic evaluation among respondents, consistent with other LMICs and similar to European OC providers [18, 23]. While CA-125 tumour marker testing was widely available, with 99% of respondents reporting access, it is best interpreted when combined with imaging and biopsy or cytology to improve the accuracy of diagnosis at low cost [24]. Multisite imaging with CT scans offers a more comprehensive assessment of disease extent and provides more accurate staging, which is essential for surgical planning, prognostication and treatment decisions. Therefore, when available, CT imaging should be utilised as a part of a standard diagnostic protocol, especially in cases of suspected metastatic disease [25].

In our study, open biopsy was the most common method of obtaining tissue for diagnosis. This contrasts with NCCN harmonised and ASCO resource-stratified guideline recommendations, which favour minimally invasive techniques. Existing data also suggest that open biopsy is associated with increased morbidity and complications [6, 7, 26]. The use of laparoscopic procedures for diagnosis in this study was uncommon, which could be attributed to the majority of respondents practicing in public hospitals, where infrastructure may be limited.

The NCCN harmonised guidelines for SSA recommend that gynaecologic oncologists perform surgery for OC if available and our survey results revealed that a majority of OC surgeries are performed by gynaecologic oncologists. However, a substantial proportion of general gynaecologists and surgeons continue to perform OC surgeries, suggesting the need for penetration of the gynaecologic oncology workforce in SSA. This gap is concerning, given the evidence suggesting improved OC outcomes among patients treated in high-volume centers by trained gynaecologic oncologists [27]. Compounding this issue, respondents also cited long surgical wait times as a major barrier to care, which was highlighted in a retrospective cohort study from Ethiopia, where nearly half of patients with advanced OC waited more than 10 weeks for cytoreductive surgery [28].

Clinical trials informing the evidence base for the surgical management of OC are typically performed in resource-rich countries with stronger health systems, skilled gynaecologic oncologists and anaesthesia expertise to perform complicated surgeries, including diaphragmatic, bowel and liver resections, in advanced OC, with the goal of achieving high rates of complete resection [15–18, 27]. Most respondents in our study (38%) reported that optimal cytoreduction was the surgical goal in their institutions, which is slightly lower than the 44% reported in an international survey of OC providers [16]. A smaller proportion of respondents in our study reported that optimal cytoreduction was not pursued for unclear reasons, despite a strong association between macroscopic residual disease and the risk of recurrence and overall survival, emphasising the importance of achieving complete cytoreduction [29, 30]. Consistent with this, a global survey of OC care found that respondents from LMICs were less likely to report <1 cm of residual disease as the surgical goal of cytoreductive surgery at their institutions [36]. Similarly, a study of recurrent OC in Ethiopia found that more than 60% of patients who experienced recurrence had residual macroscopic disease at the time of initial surgery, a finding in part attributed to limited gynaecologic oncology capacity [31].

In our study, a notable proportion of providers reported that lymphadenectomy was not available at their institution (29%), highlighting access constraints as a key barrier. Among those with access, there were inconsistencies in lymphadenectomy practices for OC patients. These findings are consistent with the survey by Barton *et al* [17] which demonstrated wide variation in lymphadenectomy practices among UK gynaecologic oncologists, with a substantial proportion of surgeons never performing pelvic or para-aortic lymphadenectomy and only a minority performing it routinely. Current guidelines recommend systematic lymph node dissection for early-stage disease and selective removal of suspicious nodes or sampling in advanced cases [6], but fewer providers in our study reported adhering to these recommendations. Systematic lymphadenectomy was underutilised in early-stage disease and inappropriately applied in some cases of advanced disease, despite evidence from the LION study showing no survival benefit and increased morbidity from extensive lymphadenopathy in advanced disease [32]. In contrast, the LION criteria were applied by 70.4% of practitioners in a survey of European OC providers [18].

A small proportion of respondents reported that surgery was pursued in recurrent OC, which has been shown to have improved survival outcomes in the DESKTOPIII and SOC-1 trials of selected patients with platinum-sensitive recurrent OC who have low-volume disease, good performance status, less than 500 mL ascites and initial complete PDS [33, 34]. Debate regarding the effectiveness of this approach persists and was the focus of a recent meta-analysis, which recommends careful patient selection with the goal of achieving complete cytoreduction to maximise potential benefit [35]. Few patients in SSA are likely to meet the criteria of initial complete cytoreduction, have low-volume disease due to advanced stage disease at presentation or have access to specialist gynaecologic services at high-volume centers where such complex surgeries could be performed [28, 31].

Collectively, the findings from this survey likely reflect the paucity of gynaecologic oncologists performing OC surgery in many SSA countries in alignment with an international survey highlighting limited surgical capacity as a significant barrier to optimal OC care in several LMICs [36]. In 2019, the International Gynaecologic Cancer Society launched the Gynaecologic Oncology Global Fellowship Program in collaboration with academic institutions in HICs- and upper-middle-income countries. The program was designed to expand the pool of trained gynaecologic oncology surgeons in LMICs. To date, several SSA nations, including Ghana, Kenya, Rwanda, Nigeria, Ethiopia, Mozambique, Zambia, Malawi and Uganda, have benefited from this initiative [37]. Enhanced multidisciplinary oversight and continued surgical skill updates, such as in minimally invasive procedures, could also be promoted to standardise care and reduce patient risk.

Platinum-taxane doublet chemotherapy was the prevalent choice for providers in the adjuvant and neoadjuvant setting, which aligns with multiple evidence-based guidelines. The role of NACT followed by IDS versus PDS in advanced OC is continually debated. In HICs, NACT and IDS are recommended for patients with stage III–IV OC who are unlikely to achieve complete cytoreduction with PDS or have high perioperative risk [8]. Approximately two thirds of respondents in our study reported use of NACT in advanced OC, which is higher than the results of an international survey, where NACT use varied by region, with higher utilisation in Europe and the United States of America [16]. This difference may reflect a later stage at presentation in many African settings and evolving practice patterns over time [16]. Emerging data suggest that the use of NACT followed by IDS is becoming increasingly common in SSA, with 33% and 42% of patients receiving NACT for OC in single institution studies in Nigeria and Ethiopia, respectively [31, 38]. A recent study in Nigeria of 126 patients revealed no difference in progression-free survival or overall survival between those who received NACT and IDS and those who received PDS [39]. The mean number of NACT cycles in our study was four; however, some respondents reported treatment beyond four cycles, despite data indicating detrimental outcomes with more than four cycles of NACT [40], suggesting guideline discordant care.

In patients with de novo or recurrent platinum-resistant OC, gemcitabine, taxanes and liposomal doxorubicin chemotherapy were most commonly used, which is consistent with current recommendations in the absence of targeted or immunotherapy options (6). Oral etoposide is considered an efficacious and cost-effective option for recurrent OC, especially in later lines of treatment [41, 42], but it was infrequently used by survey respondents.

There was low uptake of novel therapies currently indicated in adjuvant or maintenance settings in OC, such as bevacizumab and PARP inhibitors, among the survey respondents. Bevacizumab and PARP inhibitors for OC have cost-effectiveness ratios reaching up to $200,000/QALY and $150,000/QALY, respectively [43, 44]. Access to these high-cost drugs has also been identified as a barrier in OC care in a larger international survey [15]. The prohibitive costs and relatively low cost-effectiveness of these drugs limit their applicability in SSA countries with inadequate health care financing [45]. Limited access to genetic and biomarker testing, toxicity management, and modest survival benefits when weighed against cost-effectiveness may also explain why these drugs are not currently routinely used in SSA. This is exemplified in our findings, where genetic testing was unavailable to many providers, maintenance therapies were prescribed by a few and PARP inhibitors were rarely prescribed to OC patients harbouring *BRCA* mutations, findings that are consistent with observations from a study of OC patients in Nigeria [38]. These gaps align with findings of a global survey in which lack of access to genetic services for *BRCA* and homologous recombination deficiency testing was cited as a major barrier to OC care by 66% of respondents from LMICs, compared to only 8% of respondents from HICs [36].

The general lack of multidisciplinary tumour boards (MDTBs) was a major challenge highlighted by respondents, similar to the findings of a retrospective cohort study of OC patients treated in a hospital in Nigeria, where only 12% of cases were discussed in an MDTB [38]. Similarly, a global survey of OC care reported that providers from LMICs were less likely to be involved in or have MDTBs in their institution [36]. MDTBs play a critical role in maintaining guideline concordance to improve outcomes [46]. In resource-constrained settings, MDTBs can assist in both clinical decision-making and identifying context-specific treatment strategies that consider available resources [47].

Patients with locally advanced or incurable disease were infrequently referred for palliative and supportive services according to our data. Frameworks to integrate palliative care into the management of OC should be considered low-hanging fruit and enforced by policy frameworks to improve the quality of life of patients who require adequate pain control and several supportive care interventions [48].

There were several limitations to this study. First, the scope of the survey is limited by the number of questions and time required to complete the survey; for example, surveillance practices or the composition and frequency of MDTBs were not explored. Second, the number of respondents was relatively small, with the majority from Anglophone Africa; therefore, the study findings may not represent patterns of care in Francophone and Lusophone Africa. Comparatively, our study had 58 respondents from a single continent compared to 119 respondents for a global survey of OC care [15]. Third, there is potential selection bias towards providers practicing in academic and tertiary medical centers who are more likely to be members of regional professional organisations such as AORTIC, and receive and respond to the survey. The lack of a comprehensive, validated registry of cancer specialists in the region further limited our ability to define the sampling frame, assess representativeness and calculate a response rate. Additionally, we were unable to determine characteristics of survey non-responders to exclude selection bias. As a result, our findings may overrepresent resources and practice patterns in higher level centers and do not fully capture the experience of providers in community or resource-limited settings. Despite these limitations, to the best of our knowledge, this represents one of the largest assessments of its kind in the region and provides valuable insights into current practices and gaps in cancer care, which should be expanded in future studies across diverse clinical settings. Finally, comparing the clinicopathological features and survival outcomes of OC patients was beyond the scope of a survey-based study but warrants further inquiry to assess the impact of the reported practice patterns on patient outcomes.

## Conclusion

The results of this survey demonstrate areas of guideline-concordant care with respect to chemotherapy choices for OC in SSA. However, these efforts are limited by the required surgical workforce and MDTBs to provide guideline-concordant diagnoses, optimal debulking surgery and reduced access to novel therapies. Regional gynaecologic oncology surgical capacity-building efforts must be prioritised and institutions should include the adoption of contextualised resource-appropriate guidelines with evidence generated from local implementation research to guide the management of OC in SSA. Elevating the voices of oncology providers in resource-constrained settings is essential not only for identifying feasible solutions but also for aligning global oncology efforts with local priorities and realities.

## Conflicts of interest

The study authors have no conflicts of interest to declare.

## Funding

No funding received for this study.

## Author contributions

YHS, VV, RG, KW, NK, AMB, HNG, NJ, EBE and RA contributed to study design, YHS, VV, AMB, HN RG contributed to the conduct or collection, YHS, VV, POS, RG, KW and RA contributed to data analysis and interpretation, YHS, VV, RG, KW, HNG and RA contributed to the drafting of the manuscript and critical srevisions. All authors gave their final approval of the manuscript to be submitted.

## Figures and Tables

**Table 1.** Summary of providers’ challenges in the management of OC.

Theme	Subthemes	Free text responses
Surgical capacity	Limited availability of gynaecologic oncologists Limited surgeries and Poor surgical outcomes Long surgical wait times	“Unfortunately, we have only one gynaecologic oncology surgeon in the country, who really does the TAH BSO. Patients who are operated on by other surgeons undergo oophorectomy only.” “Surgeons reluctant to do cytoreductive surgery and even those that do surgery lack the expertise to do lymphadenectomy.” “We have only one oncology center” “Surgical wait time” “Late presentation with non-optimal surgery”
Disease detection and diagnostics	Presentation with advanced disease Poor awareness of signs/symptoms of OC, conflating OC symptoms with dyspepsia/gastroesophageal reflux disease Poor performance status at the time of presentation Limited access to imaging modalities such as MRI leading to suboptimal staging. Limited availability of minimally invasive techniques for tissue diagnosis (e.g. laparoscopy) and to calculate Fagotti score	“Patient reporting with very advanced/metastatic disease with peritoneal and liver deposits.” “Patients with poor ECOG >2 without histological confirmation of OC.” “Deterioration in general ECOG performance status for de novo metastatic cases hinders role of further therapy (radiation therapy or chemotherapy).” “Pelvic MRI not readily available” “Non-availability of minimally invasive technique to get tissue for diagnosis in advanced OC to allow NACT with interval surgery technique” “The absence of image guided biopsy...(lack of) the video laparoscopic device as initial tool to calculate Fagotti score”
Multidisciplinary disease team (MDT) discussions	Overall lack of tumour boards	“There is poor MDT practice” “No MDT yet”
Costs	Financial toxicity of diagnostics and treatment Costs of genetic testing	“All surgeries are expensive and done in the private sector only.” “Cost of CT/MRI imaging not affordable for most patients” “The cost and availability of targeted therapy”
Access to treatments	Targeted therapies are largely unavailable. Limited availability of *BRCA* testing Varied access to chemotherapies	“Only chemotherapy is available and occasionally bevacizumab.” “Genetic testing is not available but even if available, options for treatment based on it are not available.” “Lack of genetic testing and availability of PARP inhibitors…inaccessibility of bevacizumab” “Stock outs of chemotherapy interrupting treatment”
Tumour-specific	Resistance to multiple lines of chemotherapy especially platinum-resistance	“Managing patients with inoperable recurrent OC that is resistant to multiple chemotherapies.” “Platinum resistant epithelial OC and *BRCA*-negative patients”

## Appendix I

I.

Questionnaire distributed via AORTIC mailing lists

Question 1. What country do you practice in? (free text response)

Question 2. What is your specialisation?

- Gynaecology oncology
- Gynaecology
- Medical/Clinical oncology
- General internal medicine/Family medicine

Question 3. Do you manage OC?

- Yes
- No

Question 4. Who is responsible for financing OC care?

- Government insurance
- Private insurance
- Self-pay

Question 5. Do you use any guidelines for the management of OC?

- ASCO
- ESMO
- NCCN harmonised guidelines for SSA
- ASCO resource stratified guidelines
- Regional/national cancer guidelines
- None

Question 6. What imaging modality do you use for initial imaging to diagnose OC?

- US
- MRI
- CT scan

Question 7. Are biopsies routinely performed to confirm OC diagnosis?

- Yes
- No

Question 8. If you answered “Yes” to question #7 how are biopsies typically performed?

- Open
- Laparoscopic
- Image guided
- Blind cytology

Question 9. Is a baseline CA-125 checked prior to any intervention?

- Always
- Sometimes
- Rarely
- Never

Question 10. Do you use any scoring system (e.g Fagotti) to predict the likelihood of optimal cytoreduction during surgery?

- Yes
- No

Question 11. Who performs cytoreductive surgery?

- Gynaecology oncologist
- General surgeon
- Obstetrician/Gynaecologist
- Family practice
- Other

Question 12. At your institution, what is the goal of cytoreductive surgery?

- Complete resection (no residual disease)
- Optimal (<1 cm residual disease)
- Suboptimal (>1 cm residual disease)

Question 13. Is lymphadenectomy routinely performed with cytoreductive surgery?

- Early stage only
- Advanced stage only
- All patients
- Not available

Question 14. Do surgeons perform liver, splenic or bowel resections if indicated?

- Yes
- No

Question 15. Is debulking surgery routinely used upfront for all patients with recurrent disease?

- Yes
- No
- Only for low volume recurrent disease

Question 16. Is genetic testing performed for OC patients in your country?

- Usually
- Sometimes
- Rarely
- Never
- Not available

Question 17. Who prescribes chemotherapy?

- Gynaecology oncology
- Clinical/general oncology
- Gynaecology
- Generalist

Question 18. What is the standard initial chemotherapy regimen you most commonly prescribe for OC?

- Carboplatin/Paclitaxel
- Cisplatin/doxorubicin/cyclophosphamide
- Carboplatin/Doxorubicin
- Other

Question 19. Is single agent cisplatin prescribed as upfront therapy?

- Yes, for patients with poor performance status
- Yes, for patients with early-stage disease only
- Yes, for patients with advanced stage disease only
- Never

Question 20. What most commonly prescribed for neoadjuvant therapy?

- Carboplatin/Paclitaxel
- Cisplatin/doxorubicin/cyclophosphamide
- Carboplatin/Doxorubicin

Question 21. How many cycles of NACT do you typically give? (numerical response)

Question 22. Do you routinely prescribe VEGF inhibitors (e.g. Bevacizumab)?

- Yes, for advanced stage disease with chemotherapy
- Yes, for advanced stage disease as maintenance therapy
- Not available

Question 23. Do you routinely prescribe PARP inhibitors for *BRCA* positive patients (e.g. olaparib)?

- Yes
- No
- Not available

Question 24. What chemotherapy regimen do you mostly commonly prescribe for platinum-resistant recurrent disease?

- Taxanes
- Gemcitabine
- Oral Etoposide
- Doxorubicin/Adriamycin

Question 25. What is the most common intervention for advanced disease?

- Neo-adjuvant chemotherapy followed by surgery
- Primary surgery, followed by chemotherapy
- Surgery only
- Chemotherapy only

Question 26. How often do you refer patients with advanced OC to palliative care?

- Usually
- Sometimes
- Rarely
- Not available

Question 27. What do you find most challenging about managing OC? (free text response)
